# Educational initiatives and training for paediatric rheumatology in Europe

**DOI:** 10.1186/s12969-018-0289-y

**Published:** 2018-12-07

**Authors:** Helen E. Foster, Jelena Vojinovic, Tamas Constantin, Alberto Martini, Pavla Dolezalova, Yosef Uziel, E.M.D Smith, Lovro Lamot, Carine Wouters, Tadej Avcin, Nico Wulffraat

**Affiliations:** 1 Institute of Cellular Medicine, Faculty of Medical Sciences, Newcastle University, Newcastle upon Tyne, UK; 2 Faculty of Medicine, Department Pediatric Rheumatology, University of Nis, Nis, Serbia; 3 2nd Department of Paediatrics, Semmelweis University, Budapest, Hungary; 4 G.Gaslini Institute and University of Genoa, Genoa, Italy; 5 Paediatric Rheumatology and Autoinflammatory Diseases Centre, Department of Paediatrics and Adolescent Medicine, General University Hospital and 1st Faculty of Medicine, Charles University, Prague, Czech Republic; 6 Department of Pediatrics, Meir medical center, Tel Aviv University Sackler School of Medicine, Kfar-Saba, Israel; 7 Department of Women’s & Children’s Health, Institute of Translational Medicine, University of Liverpool, Liverpool, UK; 8 Department of Paediatric Rheumatology, Alder Hey Children’s NHS Foundation Trust, Liverpool, UK; 9 Department of Pediatrics, University of Zagreb School of Medicine, Zagreb, Croatia; 10 Division of Clinical Immunology and Rheumatology, Department of Pediatrics, Sestre Milosrdnice University Hospital Center, Zagreb, Croatia; 11 University of Leuven, Department of Microbiology and Immunology, Laboratory Immunobiology, Leuven, Belgium; 12 University Hospitals Leuven, Pediatric Rheumatology, Leuven, Belgium; 13 Department of Allergology, Rheumatology and Clinical Immunology, University Children’s Hospital, University Medical Center Ljubljana, Ljubljana, Slovenia; 14 Faculty of Medicine, University of Ljubljana, Ljubljana, Slovenia; 15 Department of Paediatrics, University Medical Center Utrecht, Utrecht University, Utrecht, Netherlands; 16 Paediatric Rheumatology, Great North Children’s Hospital, Newcastle Hospitals NHS Foundation Trust, Newcastle upon Tyne, UK

**Keywords:** Paediatric rheumatology, Training, Education, Workforce, Resources, PReS

## Abstract

The Paediatric Rheumatology European Society (PReS) has over many years, developed a portfolio of educational activities to address increasing educational needs of workforce and support young clinicians to acquire skills to develop new knowledge and deliver clinical care in the future. These educational activities aim to facilitate growth of paediatric rheumatology and ultimately improve the clinical care for children and families. This article describes the current portfolio of PReS educational activities and their relevance to the international paediatric rheumatology community.

## Introduction

The Paediatric Rheumatology European Society (PReS) has an overarching aim to promote paediatric rheumatology as a sub-specialty across Europe and also further afield through the expanding international membership. PReS aims to improve the lives of children with rheumatic diseases through collaborative networking to raise awareness, facilitate high quality research to create new knowledge, support education and training, promote advocacy and ‘best care ’ based on evidence, consensus and active consumer engagement. The importance of education and training activities to achieve these aims and address unmet need within Europe has been highlighted following the SHARE initiative (Single Hub Access for Rheumatology care, European Agency Health and Consumers, grant number 2011 1202, [ [1] Wulffraat N, 2013, [2] van Dijkhuizen EHP, 2018]) and reported in Work package 4, SHARE project, Dolezolva P (submitted for publication).

The educational activities of PReS are overseen by the PReS Education and Training Committee (ETC), currently led by one of us [TA]. The PReS ETC represents rheumatology within the European Academy of Paediatrics and has contributed to the recent 2016 European Syllabus for Training in Paediatrics (https://www.pres.eu/committee-and-wp/education.html). This new syllabus ensures that rheumatology training is compliant with the revised European Syllabus structure and format. The educational activities of PReS reflect the roles of the paediatric rheumatology specialist; namely ‘life-long’ scholarly clinician, researcher and teacher. These roles have foundations firmly embedded in the European Syllabus which sets out the minimum requirements for training to cover knowledge (*musculoskeletal conditions, multi-disciplinary team working, medications, treatment approaches*), skills *(clinical, consultation, procedural, teaching, research*) and professional roles (*managerial, leadership and mentorship*). In the context of education and training, the 2016 European syllabus for paediatric rheumatology has the following aims:

- Harmonise training programmes across European countries.
- Establish defined standards of knowledge and skills required to practice paediatric rheumatology at the tertiary care level.
- Foster development of a European network of competent specialist centres to facilitate collaborative research and training opportunities.
- Improve the clinical care of children with chronic and acute rheumatic disorders within Europe.
- Enhance European contribution to international scientific progress in the field of paediatric rheumatology.
- Promote teaching of paediatric rheumatology at undergraduate and postgraduate levels to raise awareness about the importance of early diagnosis, prompt referral to specialist care and facilitate potential recruits to fellowship programmes and the future workforce.

This document describes PReS conferences, courses, online resources programmes that are overseen by the PReS ETC (with a summary given in the Table 1) and we describe their context for the international paediatric rheumatology community. These activities are complementary to existing national training programmes (which are not discussed here further).

**Table 1:** Weblinks and Resources referred to in the manuscript

European Syllabus for Training in Paediatrics	(https://www.pres.eu/committee-and-wp/education.html
PReS website	https://www.pres.eu
Pediatric Rheumatology Online Journal	https://ped-rheum.biomedcentral.com
EULAR course information	https://www.eular.org/edu_online_course_paediatric.cfm
PMM website	http://www.pmmonline.org
PReS website – instruments and tools	http://www.pres.eu/activities/scientific-and-clinical/educational-instruments-and-tools.html
Information on Paediatric Rheumatic Diseases	https://www.printo.it/pediatric-rheumatology/
Facebook page for PReS EMERGE	www.facebook.com/PReSEMERGE,
PReS EMERGE Fellowship Programme	https://www.pres.eu/activities/young-investigators/fellowship-programs.html

### The PReS annual scientific meeting and young investigators meeting (YIM)

The annual scientific meeting of PReS disseminates advances in knowledge and research (*basic science, clinical care and education*) through ‘state of the art’ lectures and presentations. There are active allied health and consumer parallel programmes. The annual scientific meeting has become one of the most important educational events in the paediatric rheumatology international calendar and provides a platform to disseminate knowledge, opportunities to network and foster new collaborations. The annual Young Investigators Meeting (YIM) is held over the two days preceding the annual scientific meeting and is organized with support from PReS to facilitate attendance by young researchers (PhD students, post-doctoral level) and trainee doctors. The YIM aspires to nurture the PReS research ethos and aims to promote networking and foster opportunities for international collaboration; young investigators are encouraged to present their work to an international audience with feedback and guidance from PReS & YIM senior faculty. Over recent years the number of young investigators attending the YIM meeting has increased significantly with a substantial number of trainees from non-European countries (including India, Africa, North America, South America, Asia, Australia). Proceedings of future meetings and how to apply for YIM bursaries are available on the PRES website (https://www.pres.eu).

### PReS courses

The ETC oversees a ‘rolling programme’ of courses which are financially supported by grants from PReS. Details of future courses and how to register for them are available. (http://www.pres.eu). The aim is to have one of either the Basic or Advanced course per year and to reach audiences who may not otherwise have access to education and training. PReS members are encouraged to contact the ETC for further information if they wish to consider organizing a Basic or Advanced course in their country and seek PReS support.

1. The **PReS Basic courses** are set at the level of the general paediatric trainee or resident and cover the breadth of topics as outlined in the European syllabus. To date, PReS Basic Courses have been held in countries where paediatric rheumatology is less well developed Mumbai (India 2012), Sao Paulo (Brazil 2015), Budapest (Hungary 2015) and Cape Town (South Africa 2017) with future courses planned in the Ukraine and South East Asia. The aim is to promote the specialty, facilitate networking and ultimately improve patient care. The model includes an organising committee led by a local paediatric rheumatologist with faculty drawn from local and national colleagues as well as international faculty who are PReS members. Examples of previous Basic Courses are available from the ETC and it is envisaged that lectures from courses will be archived on the PReS website. The Basic Courses have a similar format (over 2–3 days with local and international faculty [*n* = 10–15]) and up to 100 delegates, with interactive lectures and workshops. Case presentations are encouraged from delegates. Workshops focused on joint examination have been included since 2015 with practical demonstrations of pGALS (paediatric Gait Arms Legs and Spine) [ [3] Foster and Jandial 2013] involving local patients. The importance of research and how to get involved is also highlighted (e.g. clinical trials, cohort studies and registries). Attendance at the Basic courses has grown year on year - e.g. Cape Town 2017 with 117 delegates, 66 from many countries in Africa and 40 from Europe and the Middle East. Feedback has been very positive to address educational needs as well as networking and peer support opportunities.

2. The **PReS Advanced courses** (e.g. JIA, Slovenia 2017, Auto-inflammatory diseases Jerusalem, Israel 2018, childhood-onset SLE Lausanne, Switzerland 2018) target a more experienced audience and in the main, senior paediatric rheumatology trainees and paediatricians with an interest in paediatric rheumatology. The childhood-onset SLE Advanced course (2018) was recorded and transmitted by video conferencing to colleagues in India, who were able to participate in case presentations and interactive sessions. It is envisaged that this facility will be available in future courses to reach a wider audience and especially those in low income countries. The scientific programme of an Advanced course is focused on a given condition (e.g. JIA, childhood-onset SLE, auto-inflammatory conditions) or specialist skills (e.g. Musculoskeletal Ultrasound [MSUS], or ‘hands on’ joint examination). The format of the Advanced Courses to date have included interactive ‘state of the art’ lectures delivered by international and local faculty, ‘meet the expert’ small group sessions, joint examination ‘how to teach’ workshops and interactive case presentations. It has been proposed by the ETC that future Advanced courses address recommendations from other PReS / EULAR initiatives such as Transitional care [ [4] Foster et al. 2016] and Musculoskeletal Ultrasound skills (MSUS) [ [5] Iagnocco 2015].

3. **PReS Specialist Skills Courses** target a more experienced audience and in the main, senior paediatric rheumatology trainees and paediatricians with an interest in paediatric rheumatology. The model of ‘pairing’ a knowledge-based Advanced Course and a Specialist Skills workshop together is likely to be ‘cost effective’ in terms of travel and time for faculty and delegates. Proposed examples include i) Advanced Course in JIA and MSUS imaging or joint examination skills and an ii) Advanced Course in JSLE or JDM and nail fold capillaroscopy .

3.1 Musculoskeletal Ultrasound (MSUS) imaging. The aim of the ETC is that all fellows in paediatric rheumatology have access to MSUS training facilitated through attending PReS courses. To date there have been MSUS courses organized with EULAR (Madrid [Spain] 2012, 2017, Belgrade [Serbia] 2013, 2015) and further courses are planned (details on PReS website). In addition to the delivery of the MSUS training courses, PReS has worked extensively with OMERACT and EULAR to work towards MSUS as a potential outcome measure in JIA with standardization of procedures, validation of definitions and grading of synovitis using MSUS in JIA. Outputs to date from these collaborations include the following:

- EULAR/PReS Recommendation for implementation of different imaging modalities in JIA [ [6] Colebatch-Bourn et al. Ann Rheum Dis 2015]
- EULAR recommendations how to organize education in MSUS [ [5] Iagnocco et al., RMD Open 2015]
- Standard scanning MSUS positions in four most affected joints in JIA [ [7] Collado 2016]
- Definition of age related vascularization and ossification of joints in children [ [8] Windschall 2017]
- Definitions of normal joint structures MSUS findings in children [ [9] Roth et al. Arthritis Care Res 2015].
- EULAR/PReS Standardized Procedures for Ultrasound Imaging in Paediatric Rheumatology (ongoing EULAR project CLI089 to produce on line web application as an educational tool)
- Preliminary Definitions for the Sonographic Features of Synovitis in Children [ [10] Roth 2017]

3.2 PReS clinical skills workshops - Joint examination skills ( [3] pGALS Foster and Jandial, pREMS [11] Foster 2011) are included in the PReS Basic Course (with a focus on pGALS) and Advanced JIA Course (with a focus on pREMS and ‘how to teach’ pGALS). Feedback to date has been very positive. The format includes ‘hands on’ workshops involving consented patients demonstrating physical signs and small groups of learners with facilitated observation and feedback from experienced paediatric rheumatologists. The ETC aims for the ‘clinical skills’ workshop concept to be expanded and to include, for example, scleroderma skin scores, nail fold capillaroscopy, muscle power testing, and transitional care consultation skills.

3.3 PReS ‘Teach the teachers’ workshops - Paediatric Rheumatologists have an integral role as educators and need to ‘reach out’ to medical students, general paediatricians and colleagues in orthopaedics and primary care to raise awareness, facilitate diagnosis and prompt referral to specialist care. Learning ‘how to teach’ is therefore an important skill and included in the European syllabus. The aim of the ETC is that all fellows will attend a ‘how to teach’ course during their training. PReS has included ‘teach the teacher’ workshops alongside the PReS scientific meetings (2016, 2017) with excellent feedback and it is envisaged that such workshops will be included in future PReS meetings and the Advanced Course rolling programme.

### PReS online resources

1. PReS Website (http://www.pres.eu) (revised and launched 2017) has signposting to available resources, conferences, courses and available bursaries.
2. Pediatric Rheumatology Online Journal – (https://ped-rheum.biomedcentral.com) founded in 2003, is a free open access journal (current impact factor of 2.328 September 2018), and supported through sponsorship from PReS. The journal has a broad range of content including clinical reviews, clinical research and basic science. The journal also publishes abstracts and proceedings from the annual scientific meetings.
3. The EULAR / PReS paediatric rheumatology online course (launched 2014) was developed with a grant from EULAR and enrolment is subsidized by EULAR with further discounted costs for low and middle income countries (https://www.eular.org/edu_online_course_paediatric.cfm). The online course is a collaborative effort between PReS and EULAR, with content written and updated yearly by senior paediatric rheumatologists with the assistance of trainees. The content is aimed at fellows in paediatric rheumatology at the start of their training, residents in adult rheumatology and paediatricians or adult rheumatologists with an interest in paediatric rheumatology. The course consists of 10 modules, each dedicated to a specific topic with a knowledge test at the end, and covering key topics as outlined in the 2016 European Syllabus in paediatric rheumatology. The course can be completed with the final online examination once a year and provides a EULAR/PReS certification for specific knowledge of paediatric rheumatology. The course has attracted more than 650 participants to date; the aim being to promote the course as the leading online education in specialist paediatric rheumatology worldwide. A EULAR/PReS published textbook to supplement the course is also now available.
4. The Paediatric Musculoskeletal Matters [PMM] website (www.pmmonline.org) can be accessed from the PReS website (http://www.pres.eu/activities/scientific-and-clinical/educational-instruments-and-tools.html). PMM was launched in 2014, is a free and open e-resource and targets medical students and family medicine doctors [ [12] Smith et al. 2016] although content is relevant to all clinicians who encounter children in their clinical practice. PMM has to date > 250,000 hits from > 150 countries. PMM International (launched September 2018) reflects paediatric rheumatology health care around the world and has contributions from an international panel of paediatric rheumatologists. PMM-Nursing is also available (launched 2017) and targets all levels of nurses involved in the care of children with rheumatic diseases. PMM is endorsed by PReS as a resource for paediatric rheumatologists and teams to use in their teaching and encompasses basic clinical skills, descriptions of normal development, the approach to investigations and initial management of musculoskeletal presentations including red flags for serious life threatening conditions. PMM is promoted as a foundation for the Basic Course and to those embarking on the EULAR/PReS online course. PMM signposts to the free pGALS app (with the 2018 version including multiple language translations) and pGALS e-module.
5. Patient and parent information - Further to the SHARE initiative there has been a complete revision of the freely available information leaflets covering a broad spectrum of conditions and including translations into several languages (https://www.printo.it/pediatric-rheumatology/). The importance of patient and family advocacy and engagement in clinical care, service delivery and research has been highlighted [ [13] Dijkhuizen et al] and includes recommendations to optimise participation and facilitate better clinical outcomes.

### PReS EMERGE fellowship programme

PReS EMERGE (EMErging RheumatoloGists and rEsearchers) encompasses young paediatric rheumatologists and researchers working together to improve clinical and research opportunities for trainees, participating in organisation of PReS educational events (YIM, Basic and Advanced courses) and liaising with other young investigator groups (e.g. EMEUNET, CARRA early investigators). The group was set up following the 2016 YIM and currently includes trainees mainly from Europe but also from all other continents. Further details are available through an active social media network (www.facebook.com/PReSEMERGE, twitter.com/PReSEMERGE) and through a bimonthly newsletter highlighting activities and opportunities for members (contact emerge.pres@gmail.com). The PReS EMERGE fellowship programme (https://www.pres.eu/activities/young-investigators/fellowship-programs.html) was launched in 2017 with financial and practical assistance for clinical trainees who are members of PReS and younger than 40 years, to facilitate placements of up to 6-months within a European Paediatric Rheumatology Centre. In addition to gaining clinical knowledge and skills, the trainee is expected to participate in a research project. From 2018, the programme is open to basic science trainees (pre-PhD to five years post-PhD) working in paediatric rheumatology. It is envisaged that the programme will enhance both clinical and basic collaborative research, foster a network of emerging and established paediatric rheumatologists and allow sharing of ideas and practices between different countries to harmonize paediatric rheumatology training.

### Other PReS educational activities

The Standards of care for Juvenile Arthritis Management in Less Resourced countries (JAMLess) is a collaborative effort with funding and support from International League Against Rheumatism (ILAR) and PReS to develop consensus based guidelines relevant to challenging health care contexts; these highlight the importance of education and training being integral to the delivery of clinical care. This project was initiated by PReS colleagues from South Africa, Argentina and India and has resulted in a consensus document [ [14] Scott C 2018], with further work to develop iterations of these recommendations for other parts of the world being underway.

## Conclusions

The activities of the PReS ETC aim to improve access to, and provision of, high quality clinical care delivered by an appropriately trained workforce and ultimately improve outcomes for children and families. The emergence of Basic and Advanced courses, specialist courses, online resources and the EMERGE fellowship programme, supplement the annual scientific meeting of PReS and YIM as a means to facilitate advances in knowledge being implemented into high quality evidence-based clinical care. We recognize that more work is needed to enable and support the expanding paediatric rheumatology community both in Europe and Internationally. The uptake and implementation of the new 2016 European syllabus into training programmes needs to address the accreditation of training centres, a proposed European sub-specialist examination and certification in paediatric rheumatology.

There is now an overarching structure to the education and training activities of the PReS ETC to address the expanding needs of the broad paediatric rheumatology community with the content based on the 2016 European Syllabus for training. The format and structure of the PReS educational portfolio, much of which is free and open to all, serves as a template relevant to the wider international paediatric rheumatology. There is clear synergy between the educational and training activities of PReS and other initiatives led by paediatric rheumatology organisations elsewhere in the world (such as those developed by ACR, CARRA, BSR, BSPAR, APRG). Through working with colleagues around the world, the aim has been to ‘reach out’, raise awareness and facilitate growth of paediatric rheumatology relevant to the local context.

## Abbreviations

ACR: American College Rheumatology
APRG: Australian Paediatric Rheumatology Group
BSPAR: British Society for Paediatric and Adolescent Rheumatology
BSR: British Society for Rheumatology
CARRA: Childhood Arthritis and Rheumatology Research Alliance
EMERGE: EMErging RheumatoloGists and rEsearchers
EMEUNET: Emerging Eular Network
ETC: Education and Training Committee
EULAR: European League Against Rheumatism
ILAR: International League Against Rheumatism
JAMLess: Juvenile Arthritis Management in Less Resourced countries
JIA: Juvenile Idiopathic Arthritis
MSUS: Musculoskeletal Ultrasound
OMERACT: Outcome Measures in Rheumatology
pGALS: paediatric Gait, Arms, Legs, Spine
PMM: Paediatric Musculoskeletal Matters
pREMS: paediatric Regional Examination of the Musculoskeletal System
PReS: Paediatric Rheumatology European Society
SHARE: Single Hub Access to Resources
SLE: Systemic Lupus Erythematosus
YIM: Young Investigator Meeting
